# A Multi-Index Fusion Adaptive Cavitation Feature Extraction for Hydraulic Turbine Cavitation Detection

**DOI:** 10.3390/e27040443

**Published:** 2025-04-19

**Authors:** Yi Wang, Feng Li, Mengge Lv, Tianzhen Wang, Xiaohang Wang

**Affiliations:** 1 Logistics Engineering College, Shanghai Maritime University, Shanghai 201306, China; 202230210063@stu.shmtu.edu.cn (Y.W.); lifeng@shmtu.edu.cn (F.L.); 202230210080@stu.shmtu.edu.cn (M.L.); 2 Harbin Electric Machinery Company Limited, Harbin 150040, China; wxhang@hec-china.com

**Keywords:** cavitation, hydraulic turbine, multi-index fusion, variational mode decomposition (VMD)

## Abstract

Under cavitation conditions, hydraulic turbines can suffer from mechanical damage, which will shorten their useful life and reduce power generation efficiency. Timely detection of cavitation phenomena in hydraulic turbines is critical for ensuring operational reliability and maintaining energy conversion efficiency. However, extracting cavitation features is challenging due to strong environmental noise interference and the inherent non-linearity and non-stationarity of a cavitation hydroacoustic signal. A multi-index fusion adaptive cavitation feature extraction and cavitation detection method is proposed to solve the above problems. The number of decomposition layers in the multi-index fusion variational mode decomposition (VMD) algorithm is adaptively determined by fusing multiple indicators related to cavitation characteristics, thus retaining more cavitation information and improving the quality of cavitation feature extraction. Then, the cavitation features are selected based on the frequency characteristics of different degrees of cavitation. In this way, the detection of incipient cavitation and the secondary detection of supercavitation are realized. Finally, the cavitation detection effect was verified using the hydro-acoustic signal collected from a mixed-flow hydro turbine model test stand. The detection accuracy rate and false alarm rate were used as evaluation indicators, and the comparison results showed that the proposed method has high detection accuracy and a low false alarm rate.

## 1. Introduction

The widespread application of renewable energy has become a core strategic consensus and universal action for the transformation of the global energy system and addressing the challenges of climate change [1]. Hydropower is a clean and renewable resource. It plays a vital role in optimizing the energy structure, ensuring energy supply security, reducing greenhouse gas emissions and promoting high-quality economic and social development [2]. As the core equipment for efficient development and utilization of hydropower resources, the performance of hydraulic turbines directly affects the operational efficiency and benefits of hydropower stations [3]. In the process of energy conversion in hydraulic turbines, various complex situations may be encountered, among which cavitation is a common occurrence that has the potential to cause serious damage to the turbine [4]. There are several issues caused by cavitation, including the weakening of equipment performance and the acceleration of material aging and damage. There may also be severe noise and vibration problems, which seriously threaten the safe and stable operation as well as the economic benefits of hydropower stations [5]. In view of this, accurate and efficient detection of cavitation phenomena in turbines has immeasurable value in extending equipment service life, improving power generation efficiency, and ensuring the overall safety of hydropower stations.

At present, many scholars have conducted relevant research on cavitation detection of hydraulic turbines. The phenomenon of cavitation can be observed through special equipment such as high-speed cameras and strobe lights. Zhang et al. [6] used a high-speed video system with high-speed camera technology to visualize the complete process of cavitation, and then performed appropriate post-processing on the obtained high-speed images to illustrate the interface topology of propeller cavitation in detail. They also detected weak tip vortex cavitation that is not easily noticeable to the naked eye through edge detection methods. 

The working principle of a stroboscope is to emit brief high-intensity light pulses to illuminate the monitored area. When cavitation occurs, the bubbles generated in the cavitation area will affect the reflection characteristics of the light, changing the intensity and characteristics of the reflected light. Therefore, by analyzing the characteristics of the reflected light received by the strobe, it is possible to further determine whether cavitation has occurred. Zhang et al. [7] employed stroboscopic imaging to systematically characterize cavitation patterns on blade surfaces, thereby investigating pressure fluctuation dynamics induced by cavitation in centrifugal pumps operating under zero inlet flow conditions. Due to the complex installation and requirement for expensive equipment, cavitation detection equipment such as high-speed cameras and stroboscopes have not been widely promoted in practical applications and have mainly been used for cavitation monitoring of model hydraulic turbines. 

In addition to observing cavitation phenomena through equipment, a large number of scholars also numerically investigated the mechanism of cavitation evolution, identified the rules of cavitation degree changes, and provided theoretical support for subsequent cavitation detection. Wang et al. [8] simulated the cavitation turbulence around a hydrofoil using a multi-scale Eulerian–Lagrangian approach to better investigate the bubble motion characteristics in macroscopic flows. On this basis, a two-way coupling Eulerian–Lagrangian method [9] was presented to analyze the cloud cavitation evolution mechanism and the internal cavity structure, and two different bubble generation mechanisms were proposed to explain the size distribution spectrum of bubbles in an unsteady cavitation flow. Wang et al. [10] also proposed an improved Eulerian–Lagrangian method to evaluate cavitation erosion. In numerical simulations of cavitation phenomena, the complex coupling of multi-scale bubble dynamics, phase transitions, and turbulence must be considered, yet practical applications often struggle to fully replicate the variable operational conditions.

In contrast, sensor-based cavitation detection methods are more common, and a large number of scholars use vibration signals [11,12,13], acoustic signals [14,15,16], pressure pulsation signals [17,18], and other devices to monitor cavitation in hydraulic turbines. Al-Obaidi et al. [19] used vibration sensors to monitor cavitation in centrifugal pumps and found that the peak amplitude of vibration signals during cavitation operation showed a more random trend compared to normal operation. This study to some extent revealed the regularity and characteristics of vibration signals after cavitation occurred, but it was not intuitive enough when applied to cavitation monitoring. 

Cavitation noises have a strong correlation with cavitation intensity. The acoustic method, especially using cavitation noise to characterize cavitation intensity, has advantages in measurements and is easy to implement, which is suitable for industrial applications [20]. Yan et al. [21] proposed a classifier model based on a support vector machine to detect cavitation using active ultrasonic flow field velocity measurements. Due to the limited installation locations of a sensor, a non-invasive installation method of acoustic signal detection is more convenient than an installation method using pressure and vibration signal analysis for some engineering applications. However, most of the methods analyze the severity of cavitation development by extracting a single feature, without verifying the robustness of these cavitation features under different operating conditions. Furthermore, these features are not leveraged for subsequent cavitation detection applications. Some studies have detected incipient cavitation, but fail to provide a secondary warning as the severity of cavitation further intensifies. Therefore, specific methods and steps have been proposed for extracting cavitation features and integrating them with actual operational data to enhance the accuracy of cavitation detection. Additionally, a secondary detection approach has been established to identify both incipient and supercavitation conditions.

The most critical step in the cavitation detection process is to denoise and extract features from the collected cavitation signals. The complexity of the operating environment of hydraulic turbines results in non-stationary and nonlinear cavitation signals collected by sensors, and the signals contain a large amount of noise interference. The commonly used signal analysis methods include time-frequency analysis methods such as short-time Fourier transform, wavelet analysis, etc. These traditional time-frequency analysis methods have poor adaptability in dealing with nonlinear and non-stationary signals, making it difficult to effectively handle the time-frequency characteristics of complex signals [22]. To accurately describe the changes in signal frequency over time, various modal decomposition algorithms have been proposed. This type of algorithm does not require pre-set basis functions; therefore, it has high adaptability and feasibility and is suitable for hydraulic turbine cavitation signals with nonlinear and non-stationary characteristics [23]. 

Sun et al. [24] decomposed the current signal during cavitation into multiple intrinsic mode functions through empirical mode decomposition (EMD) and obtained the edge spectrum of the signal through Hilbert transform to reflect the cavitation situation of a hydraulic turbine during operation. Although EMD performs well in handling nonlinear and non-stationary signals, there are also some limitations. EMD suffers from issues such as mode mixing and end effects, which can affect its accuracy and reliability in practical applications. In response to these issues, experts and scholars have proposed a series of improved algorithms, such as Ensemble Empirical Mode Decomposition (EEMD) [25], Complete Ensemble Empirical Mode Decomposition (CEEMD) [26], and Local Mean Mode Decomposition (LMD) [27]. To some extent, these problems of mode mixing and endpoint effects have been suppressed, but they cannot be completely avoided. 

Therefore, the Variational Mode Decomposition (VMD) algorithm [28] is proposed, which can decompose time series data into a series of Intrinsic Mode Functions (IMFs) with finite bandwidth and find the optimal solution of the variational modes through an iterative search. The VMD algorithm has good noise resistance and can effectively solve problems such as frequency aliasing, so it has been widely applied and studied in the field of signal processing. However, the decomposition performance of the VMD algorithm is affected by parameter selection, especially the setting of parameters such as the number of decomposition modes and penalty factors. In order to improve the decomposition performance of the VMD algorithm, a method of using an optimization algorithm to optimize parameters is proposed. Liu et al. [29] developed a hybrid optimization sparrow search algorithm to optimize the parameters of VMD, enhancing its denoising ability based on the VMD algorithm. Under low signal-to-noise ratio conditions, cavitation diagnosis of fluid machinery has been achieved. However, a combination of optimization algorithms and VMD algorithms increases the computational complexity. Therefore, a multi-index fusion adaptive VMD algorithm is proposed in this paper for detecting cavitation in hydraulic turbines.

The main contributions of this paper are as follows:

(1) A multi-index fusion VMD algorithm is proposed. This algorithm adaptively selects decomposition layers with energy and correlation as the objectives, maximizing the preservation of cavitation information.
(2) Feature selection and signal reconstruction after decomposition are conducted with reference to the frequency characteristics of incipient cavitation and supercavitation. The reconstructed signals are then utilized to extract time-frequency domain features. The detection of incipient cavitation and secondary detection of supercavitation are achieved through the proposed method.
(3) A multi-index fusion adaptive cavitation feature extraction and cavitation detection method is proposed and its performance is validated using the hydroacoustic signal collected from the hydraulic turbine model test bench.

The rest of this paper is organized as follows. The problem descriptions of the engineering case are introduced in Section 2. Section 3 presents the detailed process steps of the proposed method. The performance of the proposed method is further evaluated by industrial cases of a hydraulic turbine in Section 4. The conclusions are given in Section 5.

## 2. Problem Descriptions

### 2.1. Feature Extraction

The cavitation phenomenon of hydraulic turbines is caused by the local pressure near the blades being lower than the vaporization pressure of the liquid. Cavitation not only causes physical damage to hydraulic turbines, but also generates complex noise signals. Studying the characteristics of these noise signals is of great significance for cavitation detection. The mathematical expression of the acoustic signal collected by the hydrophone can be written in the following form:

(1) V(t)=S·[p(t)+n(t)]

where V(t) is the output of the hydrophone; S is the sensitivity of the hydrophone, representing the voltage generated per unit sound pressure; p(t) is the time-varying acoustic pressure signal; n(t) is the system noise (e.g., environmental noise, vibration noise, etc.).

According to Equation (1), it is not difficult to find that there are different types of noise, such as mechanical vibration noise and water flow noise, in the working environment of the turbine. For the cavitation detection process, the interference of these additional noises will draw the cavitation feature, resulting in the difficulty of cavitation feature extraction.

Cavitation noise refers to the noise generated during bubble formation, development, and rupture. According to the theoretical analysis of acoustic characteristics, the frequency characteristics of a single vacuole and vacuole population are basically similar, and the acoustic energy of the vacuole population radiation is roughly equivalent to the average of the radiation energy multiplied by the number of vacuoles bursting at the same time. The acoustic signal form is as follows:

(2) $$p_{1}=AU(t)e^{-\alpha t}sin(2\pi /T_{0}+\varphi )$$

where A is the amplitude, α is the attenuation index, $T_{0}$ is the pulse width, U(t) is the unit step function, and φ is the initial phase angle.

At a certain time of cavitation, the number of vacuoles bursting per unit of time is n, and the acoustic signal generated during the cavitation bursting is superimposed according to Equation (2) to obtain the cavitation noise signal at that time.

(3) $$p_{t}=\sum_{i=1}^{n}p(i)$$

In addition, the time required for complete bubble collapse can be described by the Rayleigh collapse time formula:

(4) $$T_{collapse}=0.915\cdot R_{0}\sqrt{\frac{\rho }{∆p}}$$

where $R_{0}$ is the maximum initial radius of the cavitation bubble before collapse; ρ is the liquid density; and $∆p=p_{\infty }-p_{v}$ is the driving pressure difference, defined as the difference between ambient static pressure $p_{\infty }$ and vapor pressure $p_{v}$.

The acoustic pulse width is typically defined as the time interval between the rising edge and falling edge of the acoustic pressure signal. Under ideal conditions, the pulse width is approximately equal to the collapse time:

(5) $$T_{0}\approx T_{collapse}$$

The cavitation phenomenon itself is a highly dynamic process; the generation, growth and collapse of bubbles occur at different times, the volume of vacuoles at the same time is also different, and the pulse width and amplitude are proportional to the maximum radius before the bubble collapses.

The pulse widths of the same time burst vacuoles are different, and the number of vacuoles that fail at different times is different, leading to the uneven characteristics of the cavitation noise signal in the time domain. That is, the cavitation noise signal of the hydraulic turbine is significantly non-stationary and nonlinear. Traditional linear time-invariant analysis methods find it difficult to accurately capture the dynamic features of cavitation noise signals. Existing methods for turbine cavitation feature extraction mainly extract a single feature through sensor data. However, a single feature can often only reflect some aspect of the operation state of the turbine, and it is difficult to fully capture the characteristics of the cavitation phenomenon. This may result in extracted features that are not comprehensive enough, thus affecting the robustness and accuracy of the cavitation identification.

### 2.2. Cavitation Detection

The cavitation process can be divided into non-cavitation, incipient cavitation and supercavitation according to different stages. Incipient cavitation refers to the early stage in which the local pressure on the turbine blades or other critical parts drops below the liquid vaporization pressure for the first time, leading to the formation of bubbles [30]. Although the impact of initial cavitation may not be significant for the time being, if not detected and controlled, the cavitation phenomena will evolve into more severe forms, leading to larger-scale blade erosion and performance loss. Therefore, timely detection of incipient cavitation can effectively prevent further deterioration of the cavitation phenomena and avoid irreversible damage to hydraulic turbines. Supercavitation refers to the release of enormous energy by bubbles during the collapse process, generating strong shock waves that cause erosion, fatigue cracks, and even fractures on the surface of turbine blades [31]. Supercavitation has a great destructive effect on equipment, which may lead to unplanned shutdowns and even major safety accidents. Therefore, detecting cavitation is a key measure to ensure the safe operation of equipment.

The characteristics of incipient cavitation and supercavitation may vary in the time domain, frequency domain, and time-frequency domain. The initial cavitation noise signal may be distributed throughout the entire frequency range of the collected signal, with low-frequency noise typically associated with larger bubbles and slower dynamic processes, while high-frequency noise is associated with the rapid collapse of small bubbles. During supercavitation, the rapid collapse and rupture of cavitation bubbles generates high-frequency noise, which typically ranges from a few kilohertz (kHz) to several dozen kHz. This noise is caused by the release of a large amount of energy by cavitation bubbles at the moment of collapse, resulting in high-frequency shock waves. The existing research methods on cavitation mostly focus on extracting features that can characterize the development of cavitation, and most of them have not carried out further detection work. Some scholars have also conducted tests on initial cavitation, but have not considered whether cavitation problems can still be detected when their characteristics change due to the further development of cavitation.

## 3. Multi-Index Fusion Adaptive Cavitation Feature Extraction and Detection Method

**Figure 1:**
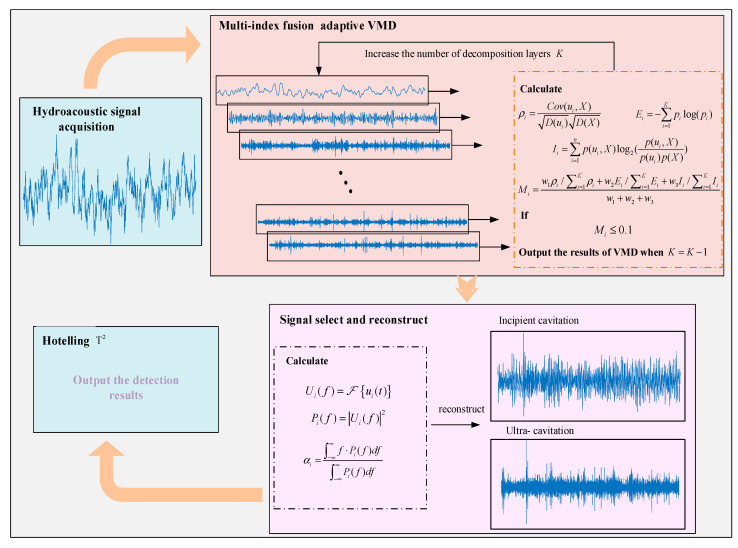
Flowchart of the multi-index fusion adaptive cavitation feature extraction and detection.

To address the aforementioned issues, a multi-index fusion-based adaptive cavitation feature extraction method, along with detection methods for incipient cavitation and supercavitation, is proposed in this paper. The difficulties in cavitation feature extraction, the inadequacy of comprehensive features, and the detection of different cavitation stages are addressed. The process of the proposed method is shown in Figure 1.

### 3.1. Multi-Index Fusion Adaptive VMD Decomposition Algorithm

The hydroacoustic signal generated by cavitation during the operation of a hydraulic turbine has nonlinear and non-stationary characteristics and is suitable for signal decomposition methods to process the signal. Based on the previous analyses, the VMD algorithm is adopted to process the collected signals. Considering the characteristics of energy changes caused by the cavitation phenomenon and considering the correlation between the decomposed signal components and the original signal, a multi-index fusion index is constructed. This indicator aims to optimize the decomposition layers of VMD, fully considering the nonlinear and non-stationary characteristics of cavitation noise. Through this optimization, it is possible to more accurately select suitable frequency band signals, thereby improving the effectiveness of feature extraction and meeting the needs of cavitation detection. The mathematical expression for the multi-index fusion index is as follows:

(6) $$M_{i}=\frac{w_{1}\rho_{i}/\sum_{i=1}^{K}\rho_{i}+{w_{2}E}_{i}/\sum_{i=1}^{K}E_{i}+{w_{3}I}_{i}/\sum_{i=1}^{K}I_{i}}{w_{1}+w_{2}+w_{3}}$$

where $\rho_{i}$ is the correlation coefficient, which reflects the correlation between the signal component and the original signal; energy entropy $E_{i}$ is the energy distribution characteristics of signal components; $I_{i}$ is the mutual information, used to measure the correlation between two random variables; $w_{1}$, $w_{2}$, $w_{3}$ are the weight of different indicators; and K is the number of decomposition layers of the signal, that is, the number of signal components. Considering the influence of each indicator on the cavitation, the weight coefficient of each indicator has been determined to be 1:2:1.

The mathematical expressions for multiple indicators are as follows:

(7) $$\rho_{i}=\frac{Cov(u_{i},X)}{\sqrt{D(u_{i})}\sqrt{D(X)}}$$

(8) $$E_{i}=-\sum_{i=1}^{K}p_{i}log(p_{i})$$

(9) $$I_{i}=\sum_{i=1}^{n}p(u_{i},X)\log_{2}\frac{p(u_{i},X)}{p(u_{i})p(X)}$$

where X is the original signal; $u_{i}$ is the *i*th component obtained after signal decomposition; and $p_{i}$ is the proportion of the *i*th component in the total energy of the signal.

(10) $$p_{i}=\frac{{u_{i}}^{2}}{\sum_{i=1}^{K}{u_{i}}^{2}}$$

**Figure 2:**
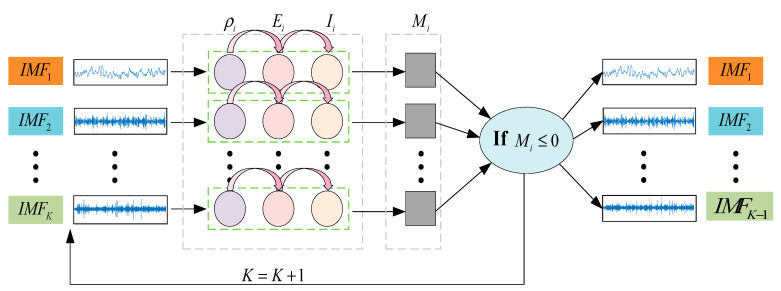
Multi-index fusion adaptive VMD process.

Performing VMD decomposition from K=2, the fusion index $M_{i}$ for each IMF component is calculated. If the values in $M_{i}$ are all greater than 0.1 [32], the number of decomposition layers is increased and recalculated. This process is continued until at least one value in $M_{i}$ is less than 0.1. At that point, the process is stopped and the decomposition results are output. The algorithm process is shown in Figure 2.

### 3.2. Signal Reconstruction and Feature Selection

The incipient cavitation noise signal may be distributed throughout the entire frequency band. Low frequency noise is usually associated with larger bubbles and slower dynamic processes, while high-frequency noise is associated with the rapid collapse of small bubbles [33,34,35]. The hydroacoustic signal of incipient cavitation is approximately distributed throughout the entire frequency range. The rapid collapse and rupture of cavitation bubbles during supercavitation can generate high-frequency noise signals, typically above 10 kHz. The IMF components are selected based on the characteristics of the incipient cavitation signal and the supercavitation signal. Firstly, the center frequency of each component is calculated. The calculation process of the center frequency is as follows:

(1) Perform Fourier transform on each component to obtain the frequency spectrum.
  (11) $$U_{i}(f)=F\left\{u_{i}(t)\right\}$$
(2) Calculate the power spectral density of the spectrum.
  (12) $$P_{i}(f)={\left|U_{i}(f)\right|}^{2}$$
(3) Calculate the center frequency using the frequency calculation equation weighted by power spectral density.
  (13) $$\alpha_{i}=\frac{\int_{-\infty }^{\infty }f\cdot P_{i}(f)df}{\int_{-\infty }^{\infty }P_{i}(f)df}$$

where $U_{i}(f)$ is the frequency spectrum of the *i*-th component, $P_{i}(f)$ is the power spectral density of the spectrum, and f is the frequency. The product integral of frequency f and power spectral density $P_{i}(f)$ is the weighted frequency.

Secondly, the IMF components obtained from decomposition are selected. Regarding the issue of detecting incipient cavitation, the components with a center frequency less than 1 kHz in the decomposed IMF components are removed, and the remaining components are linearly added to obtain the reconstructed signal for incipient cavitation detection. Regarding the issue of supercavitation detection, components with a center frequency greater than 10 kHz are selected and linearly superimposed to obtain the reconstructed signal for supercavitation detection. The reconstructed signal can be defined as:

(14) $$\hat{x}(t)=\sum_{i\in S}u_{i}(t)$$

where S denotes the index set of retained IMFs.

The reconstruction error σ(t) arises from the excluded IMFs and can be defined as:

(15) $$\sigma (t)=X(t)-\hat{x}(t)$$

The proposed reconstruction aims to eliminate low-frequency noise components (e.g., mechanical vibrations, background flow turbulence) while preserving cavitation-related signal features.

Finally, feature extraction is performed on the reconstructed signal, selecting time-frequency domain features that vary greatly with cavitation evolution, such as minimum value, rectified mean, variance, standard deviation, kurtosis, skewness, root mean square, waveform factor, peak factor, pulse factor, and margin factor, to construct a feature matrix for cavitation detection.

### 3.3. Cavitation Detection Method Based on Hotelling T^2^

Using the PCA algorithm to process the obtained feature matrix, the degree of deviation of data in principal component space, i.e., the *T*^2^ limit, is calculated to determine whether cavitation phenomenon exists. The specific steps of the proposed method are shown in Algorithm 1
**Algorithm 1** Multi-index fusion adaptive cavitation feature extraction and detection method**Input:** Hydroacoustic signal **Output:** IMFs1: Initialize decomposition layer.2: Let K=k+1.3: Perform VMD decomposition to obtain IMF component $u_{i},\;i=1,\ldots ,K$.4: Calculate the multi-index matrix $L=[\begin{array}{ccc} \rho_{1} & I_{1} & E_{1}\\ \vdots & \vdots & \vdots \\ \rho_{i} & I_{i} & E_{i} \end{array}]$ according to Equations (5)–(7).5: Calculate the fusion index matrix $M=[\begin{array}{ccc} M_{1} & \cdots & M_{i} \end{array}],\;i=1,\ldots ,K$ according to Equation (4).6: Continue the process from steps 2–6 until $M_{i}\leq 0.1$ [29], end the loop, and output the decomposition result for K=K−1.7: Calculate the center frequency $\alpha_{i}$ of each IMF.8: Select IMFs based on the center frequency and then reconstruct the signals. 9: Perform *T*^2^ testing. 

## 4. Experimental Results and Analysis

The proposed incipient cavitation and supercavitation detection methods are applied to practical hydraulic turbines in this section. Its performance in terms of accuracy and false alarm rate is also evaluated. Meanwhile, a comparison is made with existing methods to highlight the advantages and reliability of the method proposed in this paper.

### 4.1. Data Description

**Figure 3:**
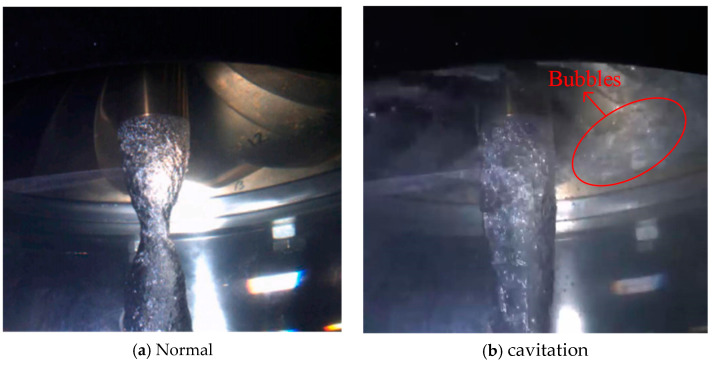
(**a**) Normal and (**b**) cavitation on hydraulic turbines.

The hydroacoustic data used in the experiment are provided by the Harbin Institute of Large Electric Machinery (HILEM). The test object is a Francis turbine, and the sensor installation position is near 0.3 D of the tailwater pipe cone. The experimental data were obtained using a B&K 8103 hydrophone produced by a well-known acoustic and vibration measurement company, Brüel&Kjær (B&K), Nærum, Denmark. with a sampling rate of 44.1 kHz. The data are a set of continuous-time signals. Figure 3 shows both normal and cavitation on Francis turbines. As shown in Figure 3, bubbles can be observed under cavitation conditions.

### 4.2. Method Performance Evaluation

The experimental verification used data from two different operating conditions. The differences between the two operating conditions are quantified and presented in Table 1. The cavitation coefficient and the degree of cavitation represented by it under each operating condition are shown in Table 2 and Table 3.

The proposed method will be used to decompose, reconstruct, extract features, and detect cavitation noise signals with different degrees of cavitation. Firstly, the data under each cavitation coefficient of each operating condition is subjected to multi-index fusion adaptive VMD decomposition. Then, IMF components for signal reconstruction according to the frequency characteristics observed at different degrees of cavitation are selected. Finally, the selected IMFs are linearly reconstructed to obtain the basic signal required for detection.

**Figure 4:**
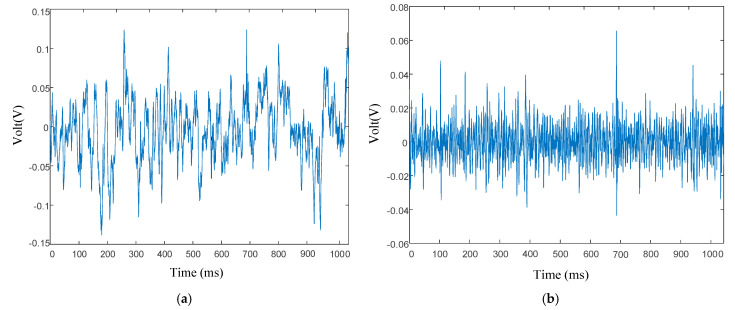
A randomly selected (**a**) original hydroacoustic signal segment and (**b**) reconstructed hydroacoustic signal segment, with a corresponding cavitation coefficient of 0.06.

Figure 4a displays a randomly selected segment of the original hydroacoustic signal, with a corresponding cavitation coefficient of 0.06. The reconstructed signal processed by the proposed method is shown in Figure 4b, where it can be observed that the reconstructed signal is essentially symmetric about the zero line, indicating the absence of DC components in the signal. The developed preprocessing algorithm has successfully removed low-frequency signal components, thereby reducing interference from low-frequency environmental noise and preventing degradation of the cavitation detection accuracy caused by DC component artifacts, which result from hydrophone baseline drift during long-term monitoring periods.

Using the sliding window method, every 45,000 data points are divided into one sample, that is, every 0.1 s is a sample. At the same time, the algorithm slides forward with a step size of 22,500 to divide the samples and obtain more samples. The time-domain and frequency-domain features of each sample are calculated to obtain a set of feature vectors, and they are combined in chronological order to obtain a feature matrix. It is input into PCA for training, constructing *T*^2^ control limits with 95% confidence, and the training and testing sets are divided in a 1:1 ratio to verify the model’s reliability. Finally, according to the continuity of cavitation on the time scale, the detection results are treated with outlier values to improve the detection efficiency.

**Figure 5:**
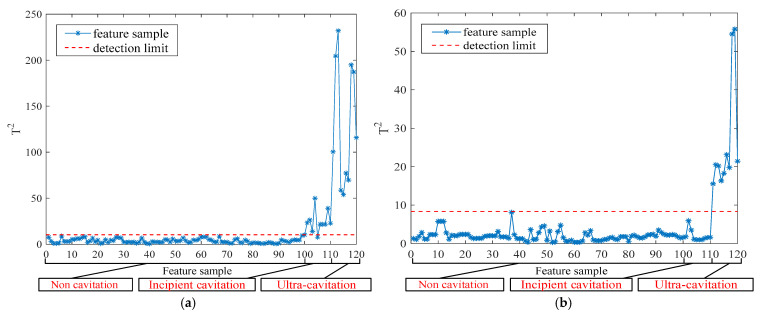
Condition 1: (**a**) Incipient cavitation detection results; (**b**) Supercavitation detection results.

**Figure 6:**
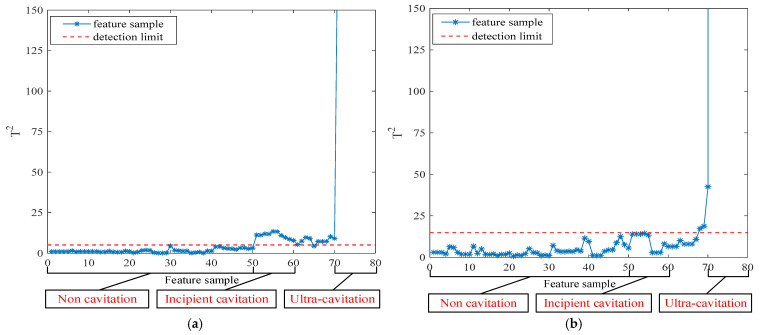
Condition 2: (**a**) Incipient cavitation detection results; (**b**) Supercavitation detection results.

The incipient cavitation detection results and supercavitation detection results under Condition 1 and Condition 2 are shown in Figure 5 and Figure 6, respectively.

From Figure 5a and Figure 6a, it can be seen that the phenomenon of incipient cavitation occurring under different operating conditions is basically detected, with only occasional missed reports. From Figure 5b and Figure 6b, it can be seen that the supercavitation phenomenon occurring under different operating conditions is completely detected.

To validate the performance of the proposed method, the Accuracy and False alarm rate were evaluated and compared with other methods to highlight its superiority. The definitions of each indicator are as follows:

$$\mathrm{Accuracy}=\frac{\text{Feature samples exceeding the detection limit}}{\text{All feature samples of cavitation}}\times 100\%\;\text{False alarm rate}=\frac{\text{Number of alarms without cavitation occurring}}{\text{The total number of alarms}}\times 100\%$$

The wavelet packet decomposition algorithm is a relatively classic signal time-frequency analysis method, often used in engineering applications to process nonlinear and non-stationary signals. Therefore, this algorithm was selected for comparison with the proposed method. In addition, using an optimization algorithm to optimize the parameters in the VMD algorithm is currently a common approach. For comparison with the proposed adaptive VMD decomposition algorithm with multi-index fusion, the Sparrow Search Algorithm (SSA) was selected in this experiment. The energy entropy was used as the objective function to optimize the VMD parameters, and the same detection algorithm was then employed for cavitation detection. Algorithms based on entropy theory can quantify the complexity of cavitation signals in hydraulic turbines without requiring additional signal processing or decomposition, thereby enabling cavitation identification. To validate the proposed approach, we adopted Multiscale Sample Entropy (MSE) as a comparative feature extraction method. Finally, as a deep learning method, Convolutional Neural Networks (CNN) exhibit exceptional performance in feature extraction and have been widely adopted across various applications. Therefore, we selected CNN as a comparative method for cavitation state detection.

The accuracy results for the proposed method and the comparative methods are shown in Table 4 and Table 5. It can be observed that the proposed method has the highest accuracy.

The false alarm rates of the proposed method and the comparative methods are shown in Table 6 and Table 7. It can be observed that the proposed method has the lowest false alarm rate.

## 5. Conclusions

A multi-index fusion cavitation feature extraction method for hydraulic turbine cavitation detection is proposed in this paper. The feasibility and accuracy of the method were verified using the experimental data provided by the Harbin Institute of Large Electric Machinery. The advantages of the proposed method are as follows:

(1) Hydroacoustic signal characteristics are analyzed and used for multi-index fusion. By employing the fusion indicator to adaptively select decomposition layers in the VMD algorithm, the impact of environmental interferences is mitigated, and cavitation information is maximally preserved. This renders the proposed method more stable and robust.
(2) Signal selection and reconstruction are utilized to unify the secondary cavitation detection process across different cavitation degrees. The cavitation detection algorithm is significantly simplified. Moreover, only basic mathematical operations are applied in the proposed method. Therefore, this method is more conducive to practical applications.
(3) The average detection accuracy of the proposed method is higher than that of the traditional wavelet packet decomposition algorithm and the SSA-VMD algorithm. The incipient cavitation can be accurately detected and a warning issued, and when cavitation further develops, another warning can be issued using the proposed method.

Considering that the proposed method in this paper utilizes the VMD algorithm, it may take a slightly longer time. An effective and real-time cavitation detection method will be pursued in future research. At the same time, in future research, we will consider cavitation detection in different parts of water turbines and pump turbines and develop more universal detection methods.

## Figures and Tables

**Table 1:** The dimensionless parameters of the two operating conditions.

Operating Conditions	Unit Rotational Speed *n*_11_	Unit Flow Rate *Q*_11_	Guide Vane Opening *a*_0_
Condition 1	74.28 r/min	1.197 m^3^/s	29.1 mm
Condition 2	83.7 r/min	1.157 m^3^/s	28 mm

**Table 2:** Data cavitation coefficient and the degree of cavitation under Condition 1.

Cavitation Degree	No Cavitation	Incipient Cavitation	Supercavitation
sigma	0.25, 0.20, 0.18, 0.15, 0.14, 0.13, 0.12, 0.11, 0.10, 0.09	0.08	0.07

**Table 3:** Data cavitation coefficient and the degree of cavitation under Condition 2.

Cavitation Degree	No Cavitation	Incipient Cavitation	Supercavitation
sigma	0.16, 0.14, 0.12, 0.10, 0.09	0.08	0.06

**Table 4:** Accuracy of incipient cavitation and supercavitation detection using different methods under Condition 1.

Method	Incipient Cavitation Detection Accuracy	Supercavitation Detection Accuracy
Wavelet packet decomposition	70%	90%
SSA-VMD	85.6%	92.4%
MSE	82.76%	93.31%
CNN	89.64%	97.91%
Multi-index fusion adaptive VMD	90%	100%

**Table 5:** Accuracy of incipient cavitation and supercavitation detection using different methods under Condition 2.

Method	Incipient Cavitation Detection Accuracy	Supercavitation Detection Accuracy
Wavelet packet decomposition	76.8%	90.2%
SSA-VMD	88.1%	96.5%
MSE	85.94%	97.26%
CNN	92.98%	100%
Multi-index fusion adaptive VMD	100%	100%

**Table 6:** False alarm rate of incipient cavitation and supercavitation detection using different methods under Condition 1.

Method	False Alarm Rate of Incipient Cavitation Detection	False Alarm Rate of Supercavitation Detection
Wavelet packet decomposition	2.3%	0.6%
SSA-VMD	1.8%	0.2%
MSE	1.94%	0.46%
CNN	1.61%	0.32%
Multi-index fusion adaptive VMD	1%	0

**Table 7:** False alarm rate of incipient cavitation and supercavitation detection using different methods under Condition 2.

Method	False Alarm Rate of Incipient Cavitation Detection	False Alarm Rate of Supercavitation Detection
Wavelet packet decomposition	1.4%	0.9%
SSA-VMD	0.6%	0
MSE	0.87%	0.12%
CNN	0.55%	0
Multi-index fusion adaptive VMD	0	0

## Data Availability

The data presented in this study are available on request from the corresponding author. The data are not publicly available due to restrictions.

## Abbreviations

The following abbreviations are used in this manuscript:

VMD	Variational Mode Decomposition
EMD	Empirical Mode Decomposition
EEMD	Ensemble Empirical Mode Decomposition
CEEMD	Complete Ensemble Empirical Mode Decomposition
LMD	Local Mean Mode Decomposition
IMFs	Intrinsic Mode Functions
PCA	Principal Component Analysis
HILEM	Harbin Institute of Large Electric Machinery
SSA	Sparrow Search Algorithm
